# What do we mean by individual capacity strengthening for primary health care in low- and middle-income countries? A systematic scoping review to improve conceptual clarity

**DOI:** 10.1186/s12960-020-00547-y

**Published:** 2021-01-06

**Authors:** Mairéad Finn, Brynne Gilmore, Greg Sheaf, Frédérique Vallières

**Affiliations:** 1 Trinity Centre for Global Health, School of Psychology, Trinity College Dublin, Dublin, Ireland; 2 Department of Public Health and Epidemiology, Royal College of Surgeons in Ireland, Dublin, Ireland; 3 UCD Centre for Interdisciplinary Research, Education and Innovation in Health Systems, School of Nursing, Midwifery and Health Systems, University College Dublin, Dublin, Ireland; 4 The Library of Trinity College Dublin, Dublin, Ireland

**Keywords:** Scoping review, Capacity strengthening, Capacity building, Low- and middle-income countries, Primary health care workers

## Abstract

**Background:**

Capacity strengthening of primary health care workers is widely used as a means to strengthen health service delivery, particularly in low- and middle-income countries. Despite the widespread recognition of the importance of capacity strengthening to improve access to quality health care, how the term ‘capacity strengthening’ is both used and measured varies substantially across the literature. This scoping review sought to identify the most common domains of individual capacity strengthening, as well as their most common forms of measurement, to generate a better understanding of what is meant by the term ‘capacity strengthening’ for primary health care workers.

**Methods:**

Six electronic databases were searched for studies published between January 2000 and October 2020. A total of 4474 articles were screened at title and abstract phase and 323 full-text articles were reviewed. 55 articles were ultimately identified for inclusion, covering various geographic settings and health topics.

**Results:**

Capacity strengthening is predominantly conceptualised in relation to knowledge and skills, as either sole domains of capacity, or used in combination with other domains including self-efficacy, practices, ability, and competencies. Capacity strengthening is primarily measured using pre- and post-tests, practical evaluations, and observation. These occur along study-specific indicators, though some pre-existing, validated tools are also used.

**Conclusion:**

The concept of capacity strengthening for primary health care workers reflected across a number of relevant frameworks and theories differs from what is commonly seen in practice. A framework of individual capacity strengthening across intra-personal, inter-personal, and technical domains is proposed, as an initial step towards building a common consensus of individual capacity strengthening for future work.

## Introduction

Capacity strengthening for primary health care workers is widely relied upon as a strategy for improving health worker performance, for strengthening health systems, and for overall quality improvement in the delivery of health services globally [1–4]. In this way, capacity strengthening interventions for human resources for health is considered an important mechanism to achieve Universal Health Coverage (UHC) under Workforce 2030, through the Global Strategy for Human Resources for Health [5, 6], and to support the implementation of the Sustainable Development Goals, articulated in Goal 17.9 [7]. In the context of these global frameworks, and in recognition of the paramount importance of a strong workforce to strengthen health systems, there are substantial investments to build primary health worker capacity in low- and middle-income countries (LMICs). Strengthening health systems, including the workforce at primary level, is thus recognised as a vehicle through which to enhance quality of care for broader populations, particularly those most vulnerable or marginalised [6].

Broadly defined as the ability to carry out stated objectives [8], capacity is a coveted aspect of human capital. Capacity *strengthening* (also commonly referred to as capacity building) has been conceptualised as an ongoing process by which individuals, groups, organisations, and societies increase their ability to perform core functions, solve problems, define and achieve objectives, and understand and deal with development needs in a broad context and sustainable manner [9]. This process is reflected across a number of prevalent models or frameworks, which consider capacity strengthening as taking place across individual, organisational, community, societal and a broader ‘systems’ components [10, 11].

Capacity strengthening for health interventions tends to take place at the level of the *individual*, ranging from training individuals in leadership, strategic thinking, supervisory, financial, project, and performance management skills, to training on more technical subjects, including laboratory, clinical and non-clinical, surveillance, monitoring, and research skills [12, 13]. It also takes place at the level of the *organisation* (i.e. through improved partnership, leadership, or governance infrastructure) [2]; and at the level of the *community* (i.e. community health programming) [14, 15]. A common inference however, is that intervening at one level (i.e. at the level of the individual primary health care worker) will subsequently result in observable changes within another level (i.e. organisational) [16, 17].

Despite efforts to map the theoretical underpinnings of capacity strengthening approaches [17], assessing or evaluating whether programmes designed to strengthen capacity effectively do so remains difficult, prompting some to call for the term to be considered and understood in more practical terms [15, 18]. Moreover, the effects of capacity strengthening interventions on improved health outcomes are difficult to ascertain. According to Crisp et al. [16], evaluating capacity must consider that (i) capacity strengthening is a process and therefore evolves over time; (ii) aggregates of individual change may be insufficient to assess organisational or community level changes, and (iii) capacity strengthening may result in unintended consequences across other components. Similarly, Aroni [18] notes that the complexity and multi-disciplinary nature of capacity strengthening in practice poses inherent challenges when it comes to measuring changes in capacity. In response to the problem of measurement, others have attempted to conceptualise capacity strengthening in terms of its indicators, or domains [15]. Nevertheless, it remains unclear what domains are most commonly used in the literature where studies claim to have demonstrated an increase in individual capacity among primary health care workers.

### Study objectives

Despite the frequent use of the term ‘capacity strengthening’ within the academic and implementation literature, and the widespread recognition of the importance of capacity strengthening in improving access to quality health care [19], there is substantial variation in how capacity strengthening is both interpreted and measured. Thus, and informed by the growing emphasis within programmes on ‘capacity strengthening’ approaches for primary health care workers and by the absence of an identified set of domains comprising ‘capacity strengthening’ at the level of the individual, the aims of this scoping review are twofold. Firstly, the study aimed to identify common domains of capacity strengthening for primary health care workers, assessed at the level of the individual. Specifically, we ask what domains are referred to, and in which combinations, in studies that aim to strengthen ‘capacity’ among primary health care workers? Second, we identify the methods used to measure and assess these domains. In achieving both of these aims, we offer insight into what is commonly understood as individual capacity strengthening for primary health care within LMICs.

## Methodology

A systematic scoping review of the literature was carried out following procedures described by Arksey and O’Malley’s [20] and Levac, Colquhoun and O’Brien [21]. Scoping review methodology was chosen because of the potential wide array of relevant literature, and because of the recognised value of scoping reviews in clarifying concepts or to investigate research conduct [22]. The current review included articles published from 2000—corresponding to the launch of the Millennium Development Goals—to October 2020. An initial search was conducted in July 2018, subsequently updated to October 2020. Both rounds of searching adhered to the same procedures, outlined below.

### Identifying relevant studies—inclusion and exclusion criteria

The population was restricted to primary health care workers, conceptualised as any non-specialist health care worker providing health care at the first point of contact to the health system, for individuals and families in the community [23, 24]. Included papers were further required to describe capacity strengthening interventions targeting the primary health care worker. Interventions specific to *research* capacity strengthening were excluded, as this has been extensively examined elsewhere [25]. Lastly, and given that capacity strengthening is recognised as a process taking place over time [16], only studies that measured changes in capacity were included. The review’s full inclusion and exclusion criteria are summarised in Table 1.

**Table 1:** Inclusion and exclusion criteria

	Inclusion criteria	Exclusion criteria
Population	Primary health care workers	Non-primary health care workers (i.e. specialists, consultants researchers)
Intervention	Interventions that aimed to strengthen capacity at the individual level	Interventions aiming to strengthen capacity at organisational or community level Interventions aiming to build research capacity
Context	Low-income and lower-middle income countries as defined by the World Bank at time of study [26]	High-income and upper-middle income countries at time of study
Outcomes	Studies must have assessed changes in capacity specifically at the level of the individual	Studies which intervened at the individual level, but which only assessed changes outside of the individual level (i.e. organisational or community)
Study design	Studies must have a comparative element assessing capacity at a minimum of two timepoints (i.e. pre–post) or using an experimental design	Non-comparative or cross-sectional designs
Date range	Published from 2000	Published prior to 2000
Publication type	Research article	Conference abstracts, conference proceedings, grey literature, report
Languages	All, though searching was primarily conducted in English	

### Identifying relevant studies—search strategy

The search terminology included four categories: capacity strengthening, health care providers, primary care, and low- and middle-income countries. A subject librarian (GS) developed search strings for six electronic databases—CINAHL, Embase, MEDLINE, ProQuest Social Science Premium Collection, PsycINFO, and Scopus—which were searched using various combinations of indexing and key search terms. Additional file 1 contains the search strings formatted for MEDLINE. The reference lists of all articles retrieved for full-text screening were also searched.

### Study selection

Two researchers (MF, BG or FV) independently assessed studies, at both title and abstract and full-text screening phase. Any discrepancies between the two primary screening authors were brought forward to the third author for review. If unresolved, all three discussed the study together and came to an agreement.

### Data management, extraction and synthesis

Returned studies were imported into EndNote, where duplicates were removed. Remaining articles were then compiled and screened in Covidence, a systematic review data management tool. A data extraction template was created in Microsoft Excel to capture information including the study characteristics, intervention characteristics, capacity strengthening domains and outcome measures. MF extracted the data for each included study and a second author (BG or FV) independently compared extractions for 20% of the articles for inter-rater reliability. The authors also discussed any cases where it was necessary to clarify decisions.

### Quality assessment

The Mixed Methods Appraisal Tool (MMAT) [27] was applied to each article. MMAT has been used successfully for other systematic literature reviews [28] and was chosen for its ability to score studies of varying methodological traditions in a single coherent manner. Due to the nature of this review, the MMAT was not used to inform the interpretation of our findings, but is presented as a resource to readers who may wish to apply individual studies to their own practice.

## Results

**Fig. 1:**
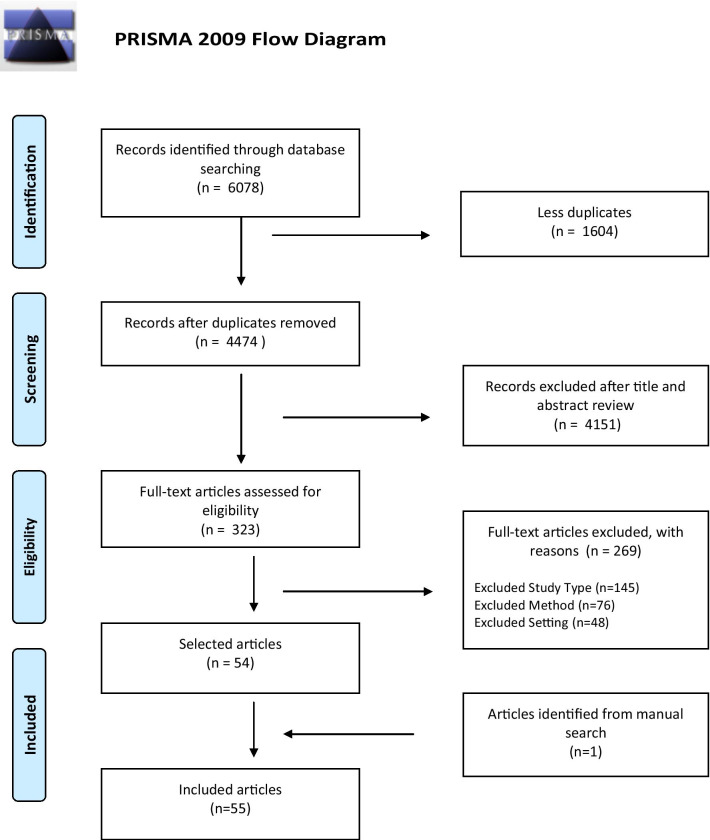
PRISMA flow diagram

In total, 6078 articles were returned from the combined database searches, of which 1604 were duplicates. Of the 4474 screened at title and abstract phase, 323 were put through for full-text screening. 269 articles were excluded at full-text phase. An additional 21 articles were identified through snowballing, of which one was included. A total of 55 articles were identified for inclusion. Figure 1 summarises the screening process.

### Article characteristics

Primary health care workers in the sample studies included physicians, nurses, midwives, medical officers, community health workers, surveillance officers, clinical officers, as well as psychiatrists and disease control officers. Forty-nine capacity strengthening interventions took place in one national setting, two were set between two- or four-country contexts; and four were set across wider regions of the Middle East, Africa and South Asia. Interventions were implemented across a range of settings including rural communities, cities, district and primary health facilities, communities, as well as conferences and educational settings. Most studies (*n* = 46) were published after 2010, with 40 published between 2015 and 2020, aligning to an increased focus on capacity strengthening resulting from the commencement of the SDGs.

Capacity strengthening interventions ranged from disease prevention and surveillance, to clinical, management and supervision interventions. Each study contained some form of training for primary health care workers with the duration of training ranging from 2.5 hours to a 4-year ongoing programme. Interventions employed an array of educational resources including manuals, activities at conferences and workshops, lectures and e-learning supports, mentorship, and case studies and role plays, overall encompassing both didactic and experiential learning. Additional file 2 contains the full table of included studies, their capacity strengthening topics and specific details on the approaches, components and measurements.

### Quality assessment

MMAT resulted in the identification of 19 studies being deemed as low quality. The majority of studies complied with the quality criteria with 14 studies meeting all five and 22 studies meeting four of five. A detailed presentation of the ratings of each of the included studies, across the five criteria, is available in Additional file 3.

### Objective 1: domains of capacity strengthening

Domains of capacity strengthening were extracted based on the terminology used in each study. Sixteen domains of capacity strengthening were identified across the sample of 55 studies: knowledge, skills, timeliness, confidence, abilities, leadership, motivation, self-efficacy, commitment, resources, attitudes, practices, competence, awareness, professional satisfaction and professional quality of life. Table 2 provides a summary of these 16 domains identified, including where they were used in combination with other domains (i.e. co-domains).

**Table 2:** Domains of individual capacity strengthening

Objective 1: domains of individual capacity strengthening		
Domain	Articles	Co-domains
Knowledge (*n* = 51)	See Additional file 4 *n* = 13 measure knowledge as the sole domain	Total with co-domains (*n* = 38) Skills (*n* = 18) For remaining, see domains below
Skills (*n* = 25)	See Additional File 4 *n* = 2 measure skills as the sole domain	Total with co-domains (*n* = 23) Knowledge (*n* = 18) For remaining, see domains below
Attitude (*n* = 11)	Hofmann-Broussard et al. [48]	Knowledge, confidence
	Kamiru et al. [45]	Knowledge, self-efficacy
	Kohrt et al. [64]	Knowledge, clinical competence
	Liautaud et al. [78]	Knowledge, skills, practice
	Minh et al. [58]	Knowledge, practice
	Sijbrandij et al. [50]	Knowledge, skills, confidence, professional satisfaction
	Spagnolo et al. [51]	Knowledge, self-efficacy, practice
	Taieb et al. [66]	Knowledge, practice
	Tharkar et al. [62]	Knowledge, behaviour
	Werdenberg et al. [72]	Practice
	Williams et al. [44]	Knowledge, self-confidence
Confidence (*n* = 8)	Bemah et al. [46]	Knowledge, skills
	Blignault et al. [73]	Knowledge, skills
	Edwards et al. [43]	Knowledge, skills, ability
	Hofmann-Broussard et al. [48]	Knowledge, attitude
	Mehrotra et al. [49]	Knowledge, self-efficacy
	Sharma et al. [42]	Knowledge
	Sijbrandij et al. [50]	Knowledge, skills, attitude, professional satisfaction
	Williams et al. [44]	Knowledge, attitude
Practice (*n* = 8)	Burnett et al. [70]	–
	Echeverri et al. [79]	Knowledge, attitude, skills
	Imani et al. [69]	Knowledge
	Liautaud et al. [78]	Knowledge, attitude, skills
	Minh et al. [58]	Knowledge, attitude
	Taieb et al. [66]	Knowledge, attitude
	Spagnolo et al. [51]	Knowledge, attitude, self-efficacy
	Werdenberg et al. [72]	Attitude
Self-efficacy (*n* = 6)	Bikinesi et al. [41]	Knowledge, professional satisfaction
	Davila et al. [47]	Knowledge, skills
	Kamiru et al. [45]	Knowledge, attitude
	Mehrotra et al. [49]	Knowledge, confidence
	Mutale et al. [39]	Knowledge, motivation
	Spagnolo et al. [51]	Knowledge, attitude, practice
Abilities (*n* = 3)	Chaoniyom et al. [53]	Knowledge, leadership, motivation
	Edwards et al. [43]	Knowledge, skill, confidence
	Sennun et al. [67]	Knowledge
Motivation (*n* = 2)	Chaoniyom et al. [53]	Knowledge, leadership, ability
	Mutale et al. [39]	Knowledge, self-efficacy
Commitment (*n* = 1)	Kim et al. [55]	Knowledge, skills, resources
Competence (*n* = 2)	Kohrt et al. [64]	Knowledge, attitude
	Mazia et al. [56]	Knowledge, skills
Leadership (*n* = 1)	Chaoniyom et al. [53]	Knowledge, ability, motivation
Resources (*n* = 1)	Kim et al. [55]	Knowledge, skills, commitment
Timeliness (*n* = 1)	André et al. [68]	Knowledge
Awareness (*n* = 1)	Oladele et al. [80]	Skills
Professional satisfaction (*n* = 1)	Bikinesi et al. [41]	Knowledge, self-efficacy
Professional quality of life (*n* = 1)	Sijbrandij et al. [50]	Knowledge, skills, attitude, confidence

Within the sample, 16 studies defined capacity strengthening as a single domain. These studies measured knowledge (*n* = 13), skills (*n* = 2) and practice (*n* = 1). Nineteen studies used a combination of two domains, 14 used three domains, and the remaining used four (*n* = 5) and five (*n* = 1). Across the full sample, the most commonly reported domain of capacity strengthening was knowledge (*n* = 51). Knowledge was most frequently strengthened alongside skills (*n* = 18), attitude (*n* = 10), confidence (*n* = 8), practice (*n* = 6) and self-efficacy (n = 6). Skills were the second most recorded domain (*n* = 25), similarly most frequently strengthened alongside knowledge (*n* = 18), followed by confidence (*n* = 4). Knowledge or skills were present in 52 of 55 studies, either alone or in combination with other domains.

Domains of capacity were strengthened across a range of aspects of primary health care. Knowledge as a sole domain was strengthened primarily on clinical and technical subjects, with a more limited number of studies focusing on management and supervision. For example, Citraningtyas et al. [29] built knowledge to enhance mental health workers’ understanding of how to provide assistance to children and adolescents in disaster affected areas in Indonesia. Strengthening of skills as the sole domain tended towards strengthening leadership and management skills among supervisors [30, 31]. In the 18 studies which measured skills alongside knowledge, capacity strengthening was conceptualised as both increases in content knowledge and observed practice, over a range of topics ranging from health promotion [32] to disease surveillance [33], and infection control [34]. One study related to ability to coach clinical skills and quality improvement [35], and another to the management of substance use conditions [36], with all other studies related to more technical skills, such as breast ultrasound interpretation [37], surveillance [33] and neo-natal continuous positive airway pressure [38].

Thirteen studies assessing the domains of self-efficacy or confidence were applied to strengthen leadership, managerial or teaching capacity [39–42]; working with patients [43–46]; and for the provision of care, such as treatment for non-communicable disease [47], anti-retroviral therapy [45], and providing mental health care [48–51], a topic which has become prevalent in more recent years.

### Objective 2: measurements of capacity strengthening

Changes in individual capacity were measured using a range of methods including pre-intervention and post-intervention objective knowledge assessments (*n* = 37), pre- and post-subjective assessments (*n *= 21), and observation or practical assessment (*n* = 20), among others. The various tools used to measure the strengthening of capacity are summarised in Table 3, according to each domain(s). Many studies contained a combination of approaches [37, 40, 46–48, 50, 52–64]. Research designs included pre- and post-survey measures, quasi-experimental, prospective and retrospective evaluations, cluster randomised trial components, and participatory needs assessments.

**Table 3:** Measurements of individual capacity strengthening

Objective 2: measurement of individual capacity strengthening		
Measurement tool	Domains	Articles
Pre–post learning assessment (*n* = 37)	Knowledge	Abrahams-Gessel et al. [52]; Ameme et al. [65]; Bemah et al. [46]; Bikinesi et al. [41]; Chaoniyom et al. [53]; Citraningtyas et al. [29]; Crocker et al. [54]; Davila et al. [47]; Garg et al. [81]; Hofmann-Broussard et al. [48]; Kabir and Hossain [82]; Kohrt et al. [64]; Mazia et al. [56]; McConnell et al. [83]; Mehrotra et al. [49]; Merchant et al. [57]; Minh et al. [58]; Murugesan et al. [74]; Namazzi et al. [84]; Okereke et al. [85]; Oleribe et al. [86]; Pringle et al. [59]; Scheel et al. [37]; Sijbrandij et al. [50]; Soeters et al. [60]; Stephens et al. [61]; Tarannum et al. [36]; Tharkar et al. [62]; Wilson et al. [63]; Yu et al. [87]
	Attitude	Kohrt et al. [64]
	Knowledge, skills	Hien et al. [32]; Weaver et al. [34]
	Awareness (clinical), skills	Oladele et al. [80]
	Knowledge, self-efficacy	Kamiru et al. [45]
	Knowledge, skills, commitment, resources	Kim et al. [55]
	Knowledge, attitude, practice	Taieb et al. [66]; Spagnolo et al. [51]
Pre–post self-report measures (*n* = 21)	Knowledge	Mehrotra et al. [49]; Sharma et al. [42]; Sennun et al. [67]
	Skills	Perrone et al. [30]
	Confidence	Bemah et al. [46]; Hofmann-Broussard et al. [48]; Mehrotra et al. [49]; Sharma et al. [42]
	Self-efficacy	Mehrotra et al. [49]; Spagnolo et al. [51]
	Knowledge, confidence	Blignault et al. [73]
	Knowledge, skills	Dawson et al. [40]
	Skills, self-efficacy	Davila et al. [47]
	Attitude; practice	Werdenberg et al. [72]
	Self-efficacy, professional satisfaction	Bikinesi et al. [41]
	Knowledge, attitude, self-confidence	Williams et al. [44]
	Knowledge, self-efficacy, motivation	Mutale et al. [39]
	Knowledge, skills, ability, confidence	Edwards et al. [43]
	Ability, leadership, motivation	Chaoniyom et al. [53]
	Attitude, confidence, professional quality of life	Sijbrandij et al. [50]
	Knowledge, attitude, practice, skills	Echeverri et al. [79]
Observation/simulation/surveillance data/practical evaluation (*n* = 20)	Knowledge	Pringle et al. [59]; Soeters et al. [60]
	Skills	Abrahams-Gessel et al. [52]; Bemah et al. [46]; Merchant et al. [57]; Namagembe et al. [31]; Scheel et al. [37]; Sijbrandij et al. [50]; Stephens et al. [61]; Tarannum et al. [36]; Wilson et al. [63]
	Ability	Sennun et al. [67]
	Practice	Burnett et al. [70], Minh et al. [58]
	Knowledge, skills	Asibon et al. [38]
	Knowledge, timeliness	André et al. [68]
	Knowledge, practice	Imani et al. [69]
	Practice, attitude	Tharkar et al. [62]
	Skills, competencies	Kohrt et al. [64]; Mazia et al. [56]
Interviews and/or focus group discussions (*n* = 3)	Skills	Crocker et al. [54]; Dawson et al. [40]
	Knowledge, skills	Kaewboonchoo et al. [33]
Hospital data/reports (*n* = 1)	Knowledge, Skills	Cosimi et al. [35]
Vignettes/case scenarios (*n* = 3)	Attitude	Hofmann-Broussard et al. [48]; Kamiru et al. [45]; Minh et al. [58]
Follow-up questionnaires 6 or 12 months later (*n* = 5)	Knowledge	Abrahams-Gessel et al. [52]; Namazzi et al. [84]; Oleribe et al. [86]
	Skills	Blignault et al. [73]
	Knowledge, skills, attitude, confidence, professional quality of life	Sijbrandij et al. [50]

Capacity strengthening for individual domains was primarily measured via objective measures (*n* = 57), either through testing of percentage increase in knowledge (*n* = 37) or through the direct observation of a specific task (*n* = 20). Change in knowledge was also assessed using subjective, self-report measures (*n* = 9), most commonly determined by asking individuals whether they had *perceived* a change in knowledge as a result of a capacity strengthening intervention. Two studies measured skills alone, one using a subjective measure [30] and the other using an objective measure [31]. For the most part, skills were measured directly through observation or practice assessment (*n* = 11), but in some cases, the acquisition of skills through practice was inferred from the acquisition of knowledge [58, 65, 66]. Ameme et al. [65] examined the impact of knowledge gained on practice and Minh et al. [58] recognised that knowledge does not always translate into practice.

Attitudes were largely measured through self-evaluation (*n* = 4) and vignettes (*n* = 3), but also through practice [62]. Confidence was always measured through pre–post self-report assessments (*n* = 8).

Twelve studies used multiple methods including a combination of subjective and objective measures of capacity. For example, Sennun et al. [67] employed a combination of subjective, self-rated assessment and objective observation to measure the effect of supervision models on the knowledge and ability of Health Officers and District Level Supervisors. In contrast, Davila et al. [47] used objective measures of knowledge and subjective, participant self-report measures to assess strengthening in the multiple domains of skills and self-efficacy for treating non-communicable diseases. Validated scales were used in 19 studies, including the Nursing Best Practice Guidelines [43]; Objective Structured Clinical Assessment (OSCA) [61]; and the mental health Global Action Programme (mhGAP) Knowledge Assessment and Attitude Scale [49–51, 64]. Full details of these validated tools are in Additional file 4.

The majority of studies (*n* = 36) measured outcomes in terms of a percentage change in the domain between pre- and post-intervention—whether objective or subjective—and the statistical significance of this change. Fourteen studies combined this with observation and six relied on observation alone to observe changes in knowledge, skills or practice [31, 38, 68–71]. In some cases, strengthened capacity through observation was rated as a percentage improvement [31], and in other cases, it was simply observed [57].

In terms of the time intervals across which capacity was assessed, most of the interventions (*n* = 28) assessed changes in capacity across short intervals, of around 1 week, ranging from 2.5 hours [57] to 2 days (n = 8) to 5 days (*n* = 8), and 6 to 8 days (*n* = 4); with others having intervals of several weeks or months (*n* = 17). These ranged from 1 month [56] up to 6 or 7 (*n* = 6) months, 18 months [72] and 4 years [43]. In four cases, domains were assessed a third time, after a period of some months, in order to assess the longer-term sustainability of the capacity strengthening intervention [52, 73]. For example, Blignault et al. [73] measured knowledge, attitudes and confidence via a pre- and post-workshop test, and measured attitudes and confidence 6 months later through a questionnaire on experience of learning in day-to-day practice. The degree or amount of capacity strengthened was primarily determined through a range of comparative and correlational analyses (i.e. *t* tests/ANOVAs, Chi-square, and regression analyses), manual content analysis, descriptive multi-variate and factor analysis, and difference-in-difference. Studies of high quality had strong conceptual frameworks and methodologies to guide the organisation and analysis of capacity strengthening.

## Discussion

A common understanding of what is meant by ‘strengthening capacity’ for the primary health care workforce is needed, given their critical role in providing quality, person-centred health care. The importance of this common understanding is further reflected through the substantial investment in and focus on building primary health worker capacity in LMICs, as a key component of health systems strengthening. This scoping review therefore sought to synthesise the existing literature to determine how the concept of individual ‘capacity strengthening’ is operationalised and measured in practice, as an initial step towards building greater consensus around what is meant by the term ‘capacity strengthening’ for primary health care workers in LMICs.

The most salient domains identified in the sample were knowledge—included in all but four studies—and skills, followed by attitude, confidence, and practice. Overall, only 20 studies in the review assessed the active implementation of objectives, through either observation or assessment of practical application. Consequently, our results suggest that the definition of capacity as “a measure of ability and, as most simply defined, capacity is the ability to carry out stated objectives” [18] is not systematically reflected in the operationalisation of capacity strengthening within the extant literature. Rather, our results suggest that capacity strengthening, where assessed, is largely operationalised as testing knowledge for action.

Capacity was predominantly measured using pre- and post-tests of knowledge change, with few studies tracing how improved knowledge translates into subsequent changes in practice. Of those studies that did assess how increased knowledge translates into practice, this was largely achieved through observation or practice assessments. Frequently, mechanisms to increase knowledge at individual level were simply *assumed* to lead to organisational-level capacity strengthening. For example, Murugesan et al. [74] built knowledge of doctors on diabetes care assuming this would lead to changes in their attitude and approach to chronic care of diseases and increase the national capacity for management of diabetes.

In addition to being deemed of higher quality, studies with more developed conceptual frameworks tended to frame capacity strengthening in broader and more comprehensive terms, that is, incorporating more than one domain and often across categories. Studies conceptualising capacity in this wider sense also tended to be informed by frameworks of capacity as broader development and aimed to strengthen each domain in a cohesive and stepwise manner. As an example, Chaoniyom et al. [53] strengthened the domain of motivation alongside knowledge, abilities and leadership. Other studies that employed broader frameworks of capacity included Davila et al. [47], who relied on experiential learning theory to inform their study design. Additionally, and while a 2017 systematic review of theoretical approaches to capacity strengthening [17] identified various theories related to capacity strengthening, we found no overlap between the review’s sample and the present sample. Overall, very few studies identified in the current study cited specific theories underpinning their approach.

Given the range of domains and measurements found in the literature, the common use of the term ‘capacity’, and the focus on strengthening health care systems within global health, we identify a need for a more defined description of what is, and what is not, considered ‘capacity strengthening’. Notably, we argue that one domain is insufficient to be considered ‘capacity strengthening’ and encourage that going forward, the use of the term ‘capacity strengthening’ be reserved for broader, multi-faceted, interventions working across more than one domain. In this way, we recommend interventions addressing a single domain to simply use the terminology ascribed to the specific activity (i.e. increasing skills or improving knowledge, etc.) as a more accurate reflection of an intervention’s impact, rather than using the broader term ‘capacity strengthening’.

**Fig. 2:**
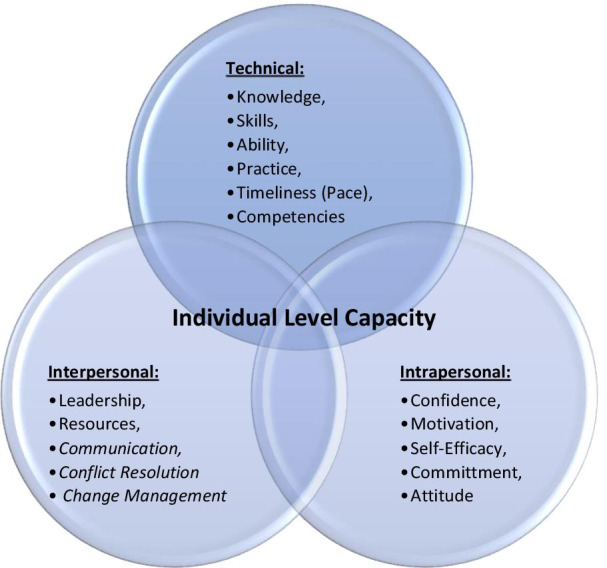
Model of individual-level capacity strengthening

In light of our findings, we propose that individual capacity be better conceptualised in terms of a combination of domains that span *technical ability,* such as knowledge and skills; *intra-personal* domains, as domains internal to the individual, such as motivation and confidence; and *inter-personal* domains, such as leadership and management. These combinations can overlap in different ways, specific to each context, to offer a more comprehensive framework. Applying this framework to the current review, all 55 studies measured technical or clinical aspects of health care, 19 measured intra-personal aspects such as confidence, self-efficacy and attitude, and two measured inter-personal aspects of leadership and resources. Figure 2 offers a summary of this framework, whereby individual capacity building is conceptualised as a broader, more comprehensive and strategic model.

The domains identified in this review therefore reflect the three domains of learning, as identified within the educational literature (i.e. cognitive, affective and psychomotor) [75–77], whereby ability, practices and skills related to a technical capacity to perform a task or set of tasks (psychomotor); motivation, self-efficacy, confidence and commitment all related to an intra-personal capacity (affective); and leadership and management related to an inter-personal capacity (cognitive). This new conceptualisation thus aligns with the literature on educational theories and other models and frameworks underpinning capacity strengthening interventions relevant to public health, for example, theories such as Diffusion of Innovations and Transformational Learning, and models such as Ecological and Interactive Systems Framework for Dissemination and Implementation [17]. We propose that initiatives to strengthen individual-level capacity and truly embed change within organisations and environments must therefore take into consideration the need to build capacity across these three spheres.

### Limitations

The current study is not without limitations. Firstly, the study was limited by having one principal data extractor, though there was discussion of any cases, where necessary. Second, while we extracted domains in terms of how they were named in the articles, we acknowledge that these different terminologies may overlap in terms of their meaning. Third, and though we did include some search terms in French and Portuguese, studies were restricted only to the English language. Fourth, setting the cut-off year at 2000 might have resulted in the exclusion of studies that demonstrate earlier conceptualisations of capacity. Finally, while this review advances our understanding of what is meant by capacity strengthening within the literature, further research is required to test the validity of our proposed framework.

## Conclusion

In the era of the Agenda 2030, Workforce 2030 and Universal Health Coverage, there is strong focus on strengthening capacities at individual, organisational and environmental levels. This study reviewed the evidence-base to identify salient domains of capacity at individual level, including how they are measured. The concept of capacity strengthening for primary health care reflected in a number of relevant frameworks and theories differs from what is commonly seen in practice, where capacity building is largely seen as knowledge increase and skills development. Conceptually however, capacity strengthening is more than knowledge acquisition. A conceptualisation of individual capacity strengthening across technical, intra-personal and inter-personal domains is proposed as a way to offer greater conceptual clarity and a more practical application of this concept in future work.

## Supplementary Information

**Additional file 1:** Example search in MEDLINE (Ebsco).

**Additional file 2:** Data extraction tables.

**Additional file 3:** MMAT quality assessment scores.

**Additional file 4:** Pre-existing validated tools.

## Data Availability

All data extracted are presented in Additional file 2. Further details can be available from the corresponding author, Mairéad Finn, upon reasonable request.
