# Supplementary material for: What do we mean by individual capacity strengthening for primary health care in low- and middle-income countries? A systematic scoping review to improve conceptual clarity
Source: Hum Resour Health. 2021 Jan 6;19:5. doi: 10.1186/s12960-020-00547-y (PMC7789571; doi:10.1186/s12960-020-00547-y)
Supplement: Supplementary file 1 — Additional file 1: Example search in MEDLINE (Ebsco). [file 12960_2020_547_MOESM1_ESM.docx]

**Additional file 1: Example Search in MEDLINE (Ebsco)**

**Search conducted was #1 AND #2 AND #3 AND (#4 OR #5 OR #6)**

| #1:  TI((capacit* OR competenc* OR capabilit*) N2 (building OR develop* OR strengthen* OR increas*)) OR AB((capacit* OR competenc* OR capabilit*) N2 (building OR develop* OR strengthen* OR increas*)) OR CI((capacit* OR competenc* OR capabilit*) N2 (building OR develop* OR strengthen* OR increas*)) |
| --- |
| #2:  MH(Developing Countries OR Asia+ OR Africa+) OR TI(((less* OR low* OR middle) N2 (income* OR resource*) N2 (countr* OR nation*)) OR “LMIC” OR “LMICs” OR Africa OR Asia OR “developing countr*”) OR AB(((less* OR low* OR middle) N2 (income* OR resource*) N2 (countr* OR nation*)) OR “LMIC” OR “LMICs” OR Africa OR Asia OR “developing countr*”) OR CI(((less* OR low* OR middle) N2 (income* OR resource*) N2 (countr* OR nation*)) OR “LMIC” OR “LMICs” OR Africa OR Asia OR “developing countr*”) |
| #3:  MH(Primary Health Care+) OR TI(((community OR district OR health OR preventative OR primary) N2 (care OR center* OR centre* OR division* OR facilit* OR health OR medic* OR post* OR team* OR unit*)) OR (package* N2 care)) OR AB(((community OR district OR health OR preventative OR primary) N2 (care OR center* OR centre* OR division* OR facilit* OR health OR medic* OR post* OR team* OR unit*)) OR (package* N2 care)) OR CI(((community OR district OR health OR preventative OR primary) N2 (care OR center* OR centre* OR division* OR facilit* OR health OR medic* OR post* OR team* OR unit*)) OR (package* N2 care)) |
| #4:  MH(Health Personnel+) OR TI((allied OR auxiliary OR community OR formal OR frontline OR health* OR hospital OR informal OR lay OR licensed OR medical OR nonprofessional OR nurs* OR peer OR social OR support OR traditional OR trained OR unlicensed OR untrained OR village OR voluntary OR welfare OR extension) N2 (aide* OR agent* OR assistant* OR attendant* OR auxiliar* OR carer* OR caregiver* OR consultant* OR distributor* OR healer* OR helper* OR individual* OR mentor* OR officer* OR person OR personnel OR practitioner* OR professional* OR promotor* OR provider* OR staff OR support OR surveyor* OR therap* OR visitor* OR volunteer* OR worker*)) OR AB((allied OR auxiliary OR community OR formal OR frontline OR health* OR hospital OR informal OR lay OR licensed OR medical OR nonprofessional OR nurs* OR peer OR social OR support OR traditional OR trained OR unlicensed OR untrained OR village OR voluntary OR welfare OR extension) N2 (aide* OR agent* OR assistant* OR attendant* OR auxiliar* OR carer* OR caregiver* OR consultant* OR distributor* OR healer* OR helper* OR individual* OR mentor* OR officer* OR person OR personnel OR practitioner* OR professional* OR promotor* OR provider* OR staff OR support OR surveyor* OR therap* OR visitor* OR volunteer* OR worker*)) OR CI((allied OR auxiliary OR community OR formal OR frontline OR health* OR hospital OR informal OR lay OR licensed OR medical OR nonprofessional OR nurs* OR peer OR social OR support OR traditional OR trained OR unlicensed OR untrained OR village OR voluntary OR welfare OR extension) N2 (aide* OR agent* OR assistant* OR attendant* OR auxiliar* OR carer* OR caregiver* OR consultant* OR distributor* OR healer* OR helper* OR individual* OR mentor* OR officer* OR person OR personnel OR practitioner* OR professional* OR promotor* OR provider* OR staff OR support OR surveyor* OR therap* OR visitor* OR volunteer* OR worker*)) |
| #5:  TI("CTCp*" OR “CHW” OR “CHWs” OR “GP” OR “GPs” OR "HEW" OR “HEWs” OR “LHW” OR “LHWs” OR “VHW” OR “VHWs” OR clinician* OR counselor* OR counsellor* OR doctor* OR doula* OR “general practitioner*” OR hospitalist* OR “linkworker” OR “link worker” OR midwi* OR nurse* OR paraprofessional* OR physician* OR physiotherapist OR psychotherapist* OR therapist*) OR AB("CTCp*" OR “CHW” OR “CHWs” OR “GP” OR “GPs” OR "HEW" OR “HEWs” OR “LHW” OR “LHWs” OR “VHW” OR “VHWs” OR clinician* OR counselor* OR counsellor* OR doctor* OR doula* OR “general practitioner*” OR hospitalist* OR “linkworker” OR “link worker” OR midwi* OR nurse* OR paraprofessional* OR physician* OR physiotherapist OR psychotherapist* OR therapist*) OR CI("CTCp*" OR “CHW” OR “CHWs” OR “GP” OR “GPs” OR "HEW" OR “HEWs” OR “LHW” OR “LHWs” OR “VHW” OR “VHWs” OR clinician* OR counselor* OR counsellor* OR doctor* OR doula* OR “general practitioner*” OR hospitalist* OR “linkworker” OR “link worker” OR midwi* OR nurse* OR paraprofessional* OR physician* OR physiotherapist OR psychotherapist* OR therapist*) |
| #6:  TI(“Activista” OR “Agente comunitario de salud” OR “Agente comunitario de saude” OR “Anganwadi” OR “Animatrice” OR “Brigadista” OR “Colaborador voluntario” OR “Community drug distributor” OR “Monitora” OR “Mother coordinator” OR “Outreach educator” OR “Promotora” OR “Rural health motivator” OR “Shastho shebika” OR “Shastho karmis” OR “Sevika” OR “Village drug-kit manager”) OR AB(“Activista” OR “Agente comunitario de salud” OR “Agente comunitario de saude” OR “Anganwadi” OR “Animatrice” OR “Brigadista” OR “Colaborador voluntario” OR “Community drug distributor” OR “Monitora” OR “Mother coordinator” OR “Outreach educator” OR “Promotora” OR “Rural health motivator” OR “Shastho shebika” OR “Shastho karmis” OR “Sevika” OR “Village drug-kit manager”) OR CI(“Activista” OR “Agente comunitario de salud” OR “Agente comunitario de saude” OR “Anganwadi” OR “Animatrice” OR “Brigadista” OR “Colaborador voluntario” OR “Community drug distributor” OR “Monitora” OR “Mother coordinator” OR “Outreach educator” OR “Promotora” OR “Rural health motivator” OR “Shastho shebika” OR “Shastho karmis” OR “Sevika” OR “Village drug-kit manager”) |
