# Supplementary material for: What do we mean by individual capacity strengthening for primary health care in low- and middle-income countries? A systematic scoping review to improve conceptual clarity
Source: Hum Resour Health. 2021 Jan 6;19:5. doi: 10.1186/s12960-020-00547-y (PMC7789571; doi:10.1186/s12960-020-00547-y)
Supplement: Supplementary file 3 — Additional file 3: MMAT quality assessment scores. [file 12960_2020_547_MOESM3_ESM.docx]

**Quality Appraisal Notes**

1. All studies in our review are empirical and therefore we did not use the two screening questions as screens. Rather we used them to collate the relevant information.
2. The Quality Appraisal is of the overall method of the paper, not just that which relates to capacity strengthening.

| **1** | | | **Study ID: 1338**  **Author and Year: Abraham-Gessel (2015)**  **Study Design: Mixed Methods**  **Overall Score: 5** | | |
| --- | --- | --- | --- | --- | --- |
| **Q** | | | **Ans.** | | **Comment** |
| S1 | | | Yes | | To quantitatively assess the performance of CHWs during training and to qualitatively capture their training and fieldwork experiences while conducting non-invasive screening for cardiovascular disease (CVD) risk in their communities. |
| S2 | | | Yes | | Written tests and focus group discussions |
| 1 | | | Yes | | To capture both performance and experience to enhance understanding. |
| 2 | | | Yes | | Results from the software analyses and the manual analyses were combined for a comprehensive assessment of the data. |
| 3 | | | Yes | | Overall the results demonstrate that CHWs can be effectively trained to conduct non-invasive screening for CVD in the communities where they reside and that new knowledge acquired during training persists for up to six months after the completion of screening. The success of the training is dependent on the incorporation of expert and intimate knowledge of the communities themselves, both in the design and implementation phases of training. |
| 4 | | | Yes | | There are no divergences to report |
| 5 | | | Yes | | Numbers taken into consideration for statistical tests, and quality analysis for qualitative data. |
| **2** | | **Study ID: 3818**  **Author and Year: Al Shdaifat et al (2019)**  **Study Design: Cohort Study – Quantitative Non-Randomised**  **Overall Score: 4** | | | |
| S1 | | Yes | | To enhance the primary care knowledge and skills of GPs providing primary healthcare in the outpatient clinics serving Palestinian refugees in Jordan with a low-cost model that had minimal impact on patient care loads. | |
| S2 | | Yes | | Needs Assessment, Pre knowledge and post knowledge assessments and clinical checklists, participant satisfaction | |
| 1 | | Yes | | The design is accounted for in describing the structure of the program. | |
| 2 | | Yes | | The components are sufficiently integrated | |
| 3 | | Yes | | The outputs are adequately interpreted | |
| 4 | | Yes | | They are fully accounted for in the presentation of findings and discussion | |
| 5 | | No | | There is insufficient detail | |
| **3** | | | **Study ID: 809**  **Author and Year: Ameme et al (2016)**  **Study Design: Quantitative** **Descriptive**  **Overall Score: 5** | | |
| S1 | | | Yes | | To evaluate the gain in knowledge by participants |
| S2 | | | Yes | | Evaluation of the training was done through pretest posttest, feedback from participants during and after the training, and through the quality of written and oral training-related assignments. |
| 1 | | | Yes | | Full class of participants |
| 2 | | | Yes | | Full class of participants |
| 3 | | | Yes | | The participants’ assessment of various aspects of the training workshop was done by using a five point Likert-type scale, whose responses ranged from “poor” to “excellent”. |
| 4 | | | Yes | | Response Rate 93.94% from a defined population |
| 5 | | | Yes | | Descriptive |
| **4** | | | **Study ID: 520**  **Author and Year: André (2017)**  **Study Design: Quantitative Descriptive**  **Overall Score: 0** | | |
| S1 | | | Yes | | Field Epidemiology Training Program, “learning by doing” approach  Increases participants’ capacity to apply epidemiologic concepts while strengthening the health system through the production of useful epidemiologic field products that provide information for decision making  Improving quality of surveillance and ability to use surveillance data  Process and early results of FETP-Frontline |
| S2 | | | Yes | | Proportion of graduates and timeliness of reporting |
| 1 | | | CT | | The article does not have much on methodology. Mostly description of overall programme, including outcomes.  Persons trained were purposefully selected based on their job description, however additional cadres of health workers were also eligible for training. Those trained then became the study participants. |
| 2 | | | CT | | It appears that all those trained were included as participants, with results of the training including all persons. However, this is not explicitly noted. |
| 3 | | | CT | | There is limited detail on the specific methods. |
| 4 | | | CT | | There are no detail provided to support this category. |
| 5 | | | CT | | There are limited details provided to support this category. |
| **5** | | **Study ID: 3883**  **Author and Year: Asibon et al (2020)**  **Study Design: Mixed Methods**  **Overall Score: 3** | | | |
| S1 | | Yes | | To evaluate whether peer mentorship was an effective and sustainable way of improving and maintaining knowledge and skills on neonatal continuous positive airway pressure (CPAP) in a low‐resource setting with a high turnover of healthcare providers | |
| S2 | | Yes | | Data was collected through observation and self-assessment | |
| 1 | | No | | No clear rationale is provided | |
| 2 | | Yes | | There is sufficient integration to answer the research question | |
| 3 | | Yes | | The outputs are adequately interpreted | |
| 4 | | Yes | | They are accounted for in the discussion and in the limitations | |
| 5 | | No | | There is insufficient detail | |
| **6** | | **Study ID: 3784**  **Author and Year: Bemah et al (2019)**  **Study Design: Quantitative Descriptive**  **Overall Score: 2** | | | |
| S1 | | Yes | | To strengthen local capacity and combat disease transmission, various healthcare worker (HCW) trainings, including the Ebola treatment unit (ETU) training, safe & quality services (SQS) training and rapid response team (RRT), were developed and implemented between 2014 and 2017. | |
| S2 | | Yes | | Data from the ETU, SQS and RRT trainings were analyzed to determine knowledge and confidence gained. | |
| 1 | | CT | | The analysis sample was limited to individuals who completed both pre- and post-tests. | |
| 2 | | CT | | No details are provided | |
| 3 | | Yes | | Measurements relate to knowledge and skills gained | |
| 4 | | CT | | No data on response rates and number of participants for assessment is included. | |
| 5 | | Yes | | Results of the statistical tests were reported with the corresponding mean changes and 95% confidence intervals. A p-value < 0.05 was considered statistically  significant. | |
| **7** | | **Study ID: 3867**  **Author and Year: Bikenisi et al (2020)**  **Study Design: Mixed Methods**  **Overall Score: 4** | | | |
| S1 | | Yes | | To assess a pilot of the first HIV Project ECHO (Extension for Community Health Outcomes) | |
| S2 | | Yes | | Methods included pre/post program assessments of healthcare worker knowledge, self-efficacy, and professional satisfaction; assessment of continuing professional development (CPD) credit acquisition; and focus group discussions and in-depth interviews. | |
| 1 | | No | | No clear rationale for employing mixed methods is provided | |
| 2 | | Yes | | The findings are presented separately but integrated in the discussion | |
| 3 | | Yes | | The interpretation in the discussion is strong | |
| 4 | | Yes | | These are accounted for in the discussion | |
| 5 | | Yes | | There is sufficient detail on each approach | |
| **8** | | | **Study ID: 1815**  **Author and Year: Blignaut et al (2012)**  **Study Design: Mixed methods**  **Overall Score: 3** | | |
| S1 | | | Yes | | Evaluation Forms to all Members. (Also cites qualitative evaluation findings)  Participants completed a self-evaluation, a questionnaire assessing attitudes towards people with mental illness (the MICA scale), and a course evaluation. Six months later, a mailed questionnaire asked about experiences applying learning in day-to-day practice. |
| S2 | | | Yes | | Assess attitudes to mental illness, evaluate course, and learn of experiences |
| 1 | | | No | | A clear rationale is not explicated, the methods were just presented |
| 2 | | | No | | They are all presented to but not necessarily integrated strategically |
| 3 | | | Yes | | Scale to self-report knowledge gain and confidence to apply in practice (every day experiences?) |
| 4 | | | Yes | | There are no divergences to report |
| 5 | | | CT | | It is impossible to tell |
| **9** | | **Study ID: 4242**  **Author and Year: Burnett et al (2018)**  **Study Design: Mixed Methods**  **Overall Score: 4** | | | |
| S | | Yes | | Within the Integrated Infectious Diseases Capacity Building Evaluation, the study sought to compare training alone and training combined with on-site support (OSS). | |
| S2 | | Yes | | Clinical faculty assessed MLP HIV clinical practice on 6 tasks. | |
| 1 | | Yes | | Health facilities were assigned to the 2 study arms after baseline data collection (1:1 balance) by stratified random selection, with stratification for 2 on-site interventions that would have potentially contaminated the trial. | |
| 2 | | Yes | | Randomization was performed using random number generation in Stata 10.1 after the completion of the baseline assessment. | |
| 3 | | Yes | | Two clinical faculty that participated at baseline left their positions and were replaced at endline. | |
| 4 | | No | | This study was not blinded during the intervention or endline assessments. | |
| 5 | | Yes | | Two participants did not complete due to illness. | |
| **10** | | | **Study ID: 2896**  **Author and Year: Chaoniyom et al (2005)**  **Study Design: Mixed Methods (Quantitative Non-Randomised and Qualitative)**  **Overall Score: 4** | | |
| S1 | | | Yes | | To strengthen FHLs’ capabilities for sustaining community based health promotion and network establishment (and to measure the improvement) |
| S2 | | | Yes | | A quasi-experimental, One-Group, PreTest/Post-Test design; observation checklists and discussion guidelines |
| 1 | | | Yes | | A full account of the purpose of the range of methods used is given in the section ‘materials and methods’. |
| 2 | | | Yes | | They are fully integrated in a conceptual diagram and in the discussion |
| 3 | | | Yes | | They are interpreted sequentially but with a logical flow and as part of a coherent whole |
| 4 | | | Yes | | There are no divergences to report |
| 5 | | | No | | The quantitative component is, but not the qualitative component. |
| **11** | | | **Study ID: 77**  **Author and Year: Citraningtyas et al (2017)**  **Study Design: Mixed Methods**  **Overall Score: 4** | | |
| S1 | | | Yes | Describes lessons learned from mental health workers who participated in Capacity Building for Child and Adolescent Mental Health in Disaster Areas (CAMHD) training of trainers in Jakarta. This training module aimed to enhance mental health workers’ understanding of how to provide relevant assistance to children and adolescents in disaster affected areas. | |
| S2 | | | Yes | Data were collected from the training participants in the form of pre and post tests, questionnaires demographic data, training evaluation forms, and focus group discussions. | |
| 1 | | | Yes | It states that mixed method studies have been demonstrably useful for research into working in complex emergencies such as disasters (Bolton, Tol & Bass, 2009). | |
| 2 | | | Yes | They are integrated in the results and discussion sections | |
| 3 | | | Yes | They are interpreted in great detail | |
| 4 | | | Yes | There are no divergences reported | |
| 5 | | | No | Some details are given but not a full account | |
| **12** | | | **Study ID: 1194**  **Author and Year: Cosimi (2015)**  **Study Design: Quantitative Non-Randomised**  **Overall Score: 3** | | |
| S1 | | | Yes | | A prospective evaluation of the quality of care in seven HIV clinics before and after implementation of the coaching activities by the PCT |
| S2 | | | Yes | | Performance measurement and quality improvement. |
| 1 | | | CT | | There is no detail about randomisation or representation. A sample of charts were selected. |
| 2 | | | Yes | | There were core indicators to measure quality of care |
| 3 | | | Yes | | The study reports outcome data aligned to their research objectives. |
| 4 | | | No | | There is little presentation of potential cofounders. This study looks at performance of six hospitals, but does not provide any analysis between to understand differences based on cofounders or other context attributes. |
| 5 | | | Yes | | It appears the intervention was run accordingly. |
| **13** | | | **Study ID: 1230**  **Author and Year: Crocker (2016)**  **Study Design: Qualitative**  **Overall Score: 5** | | |
| S1 | | | Yes | | To evaluate training in WaSH |
| S2 | | | Yes | | Qualitative interviews and development of a conceptual framework for evaluating training in WaSH. |
| 1 | | | Yes | | It explored the fact that the training program resulted in trainees learning the CLTS process and new skills, and improving their individual performance through application of advocacy, partnership, and supervision soft skills. |
| 2 | | | Yes | | Interviews with 42 government officials |
| 3 | | | Yes | | Clear extrapolation of findings from data |
| 4 | | | Yes | | Sufficient use of quotes and transparency of counts |
| 5 | | | Yes | | There is coherence across data sources, collection, analysis and interpretation |
| **14** | | | **Study ID: 1186**  **Author and Year: Davila et al (2014)**  **Study Design: Mixed Methods**  **Overall Score: 3** | | |
| S1 | | | Yes | | Describe the design and outcomes of applied training in NCD epidemiology and control piloted in Tanzania |
| S2 | | | Yes | | Kirkpatrick’s evaluation model for measuring reactions, learning, behaviour and results using pre- and post-tests and closed-ended and open-ended questions. |
| 1 | | | No | | No rationale is provided for the range of methods. The range of methods is broad (5). |
| 2 | | | Yes | | All methods are interpreted cohesively under ‘trainees’, ‘reaction and learning’ and ‘behaviour and results’ |
| 3 | | | Yes | | As above, and in the discussion |
| 4 | | | Yes | | There are no divergences to report |
| 5 | | | No | | Sufficient details for quantitative but not for qualitative |
| **15** | | | **Study ID: 1226**  **Author and Year: Dawson et al (2016)**  **Study Design: Mixed Methods**  **Overall Score: 4** | | |
| S1 | | | Yes | | An evaluation of the Maternal Child Health Initiative (MCHI) (2012–2013) to determine key factors contributing to maternal health workforce strengthening. |
| S2 | | | Yes | | Data were gathered through interviews, focus group discussions and surveys with clinicians, midwifery students and staff from nursing and midwifery schools and National Department of Health staff. Documentation from stakeholder meetings  and regular site reports were reviewed. |
| 1 | | | Yes | | To provide multiple perspectives on the outcomes of interest. |
| 2 | | | Yes | | Each data set was analysed separately and meta-inferences were drawn across all data. |
| 3 | | | Yes | | 9 themes are presented in the findings, drawing on all data sources. |
| 4 | | | Yes | | There are no divergences to report |
| 5 | | | No | | Some detail is provided but not a full extensive account. |
| **16** | | **Study ID: 4121**  **Author and Year: Echeverri et al (2018)**  **Study Design: Mixed Methods**  **Overall Score: 4** | | | |
| S1 | | Yes | | To decrease the treatment gap of mental, neurological and substance use (MNS) conditions | |
| S2 | | Yes | | Data acquired through results of a process evaluation | |
| 1 | | Yes | | The rationale for the various approaches is provided | |
| 2 | | Yes | | The findings are presented separately but integrated in the discussion | |
| 3 | | Yes | | The interpretation in the discussion is strong | |
| 4 | | Yes | | These are accounted for in the discussion | |
| 5 | | No | | There is not enough detail to address this requirement | |
| **17** | | | **Study ID: 1208**  **Author and Year: Edwards et al (2016)**  **Study Design: Quantitative Non-Randomised Control Trial**  **Overall Score: 4** | | |
| S1 | | | Yes | | Determine the impact of establishing multi-stakeholder leadership hubs on evidence-informed HIV care practices |
| S2 | | | Yes | | Prospective quasi-experimental study |
| 1 | | | Yes | | Stratified, multi-stage random sampling of participants |
| 2 | | | Yes | | The measurements are clearly defined and appropriate. |
| 3 | | | Yes | | Full provision of outcome data in Table 7 |
| 4 | | | CT | | Details not contained |
| 5 | | | Yes | | There were some slight modifications (e.g.) timing of activities, but nothing more substantial as detailed in the MMAT. |
| **18** | | **Study ID: 3821**  **Author and Year: Garg et al (2020)**  **Study Design: Mixed Methods**  **Overall Score: 4** | | | |
| S1 | | Yes | | To train medical doctors as future trainers and developing competency-based skills for each component of a Maternal Death Surveillance and Response program. | |
| S2 | | Yes | | A training evaluation study was carried out on 144 workshop participants. Workshop outcomes in terms of trainees’ knowledge were assessed using a 20-item pre-post questionnaire and skill evaluations through group work. | |
| 1 | | Yes | | A full account is provided in the set up of the study | |
| 2 | | Yes | | The components are sufficiently integrated | |
| 3 | | Yes | | The outputs are adequately interpreted | |
| 4 | | Yes | | They are fully accounted for in the presentation of findings and discussion | |
| 5 | | No | | Some details are omitted | |
| **19** | | | **Study ID: 2599**  **Author and Year: Hien et al (2008)**  **Study Design: Quantitative Randomised Control Trial**  **Overall Score: 4** | | |
| S1 | | | Yes | | To evaluate the effectiveness of an educational program entitled ‘Capacity building for community leaders in a healthy living environment,’ and to assess the usefulness of a participatory style of education and the applicability of an intersectoral approach in the educational process. |
| S2 | | | Yes | | Randomized controlled study of a 5-day participatory-style educational program. |
| 1 | | | Yes | | Detailed on page 356 |
| 2 | | | Yes | | There were no significant differences with respect to any of the variables |
| 3 | | | Yes | | Detailed in Tables 2 and 3 |
| 4 | | | CT | | Details not contained |
| 5 | | | Yes | | There is no account of deviation from adhering to the assigned intervention |
| **20** | | | **Study ID: 259**  **Author and Year: Hofmann – Broussard et al (2016)**  **Study Design: Quantitative Non- Randomised Control Trial**  **Overall Score: 3** | | |
| S1 | | | Yes | | To acquire knowledge and skills to be able to recognize, refer and support people experiencing mental disorders. |
| S2 | | | Yes | | A pre-test post-test controlled trial design |
| 1 | | | Yes | | Participants were members of the training course |
| 2 | | | Yes | | The study used vignettes and validated scales to measure mental health literacy, stigma, and self-confidence |
| 3 | | | CT | | I OR Outcome data is fully presented in the tables? |
| 4 | | | No | | No details are given of confounders |
| 5 | | | Yes | | The mental health program is administered as intended |
| **21** | | | **Study ID: 1117**  **Author and Year: Imani et al (2015)**  **Study Design: Quantitative Randomised Control Trial**  **Overall Score: 4** | | |
| S1 | | | Yes | | To evaluate the effects of two interventions, Integrated Management of Infectious Disease (IMID) training and on-site support (OSS), on clinical practice of mid-level practitioners. |
| S2 | | | Yes | | Randomised Control Trial |
| 1 | | | Yes | | Thirty-six eligible health facilities were selected from all major regions of Uganda [26] and randomized as clusters. Eighteen health facilities were then randomly assigned to  receive OSS (arm A) in 2010 while the other 18 facilities received it in 2011 and served as a control for OSS during the trial (arm B). |
| 2 | | | Yes | | Patient samples were comparable across arms at baseline and endline. |
| 3 | | | Yes | | See Figure Two |
| 4 | | | No | | This study was not blinded after randomization in February 2010. |
| 5 | | | Yes | | Participants did adhere |
| **22** | | **Study ID: 4136**  **Author and Year: Kabir and Hossain (2019)**  **Study Design: Quantitative Non Randomised**  **Overall Score: 2** | | | |
| S1 | | Yes | | To evaluate changes in knowledge of primary and community level healthcare providers about cardinal signs, course of leprosy treatment, and drug use for paucibacillary (PB) and multibacillary (MB) leprosy cases | |
| S2 | | Yes | | Two surveys before and after the intervention | |
| 1 | | No | | Sampling strategy was purposive with no rationales provided | |
| 2 | | Yes | | Detailed survey provided covering key aspects of intervention aims | |
| 3 | | Yes | | Outcome data presented on all key aspects of survey and assessment | |
| 4 | | No | | Difference in size between baseline and endline. Only assessed workforce type, no other confounders (i.e. age, gender, experience) | |
| 5 | | CT | | No clear details on implementation; difference in baseline and endline in terms of numbers for evaluation | |
| **23** | | | **Study ID: 2215**  **Author and Year: Kaewboonchoo …. (2011)**  **Study Design: Quantitative Description**  **Overall Score: 5** | | |
| S1 | | | Yes | | Evaluate community-based participation in the process of capacity building in the areas |
| S2 | | | Yes | | Needs assessment, a workshop and a training program |
| 1 | | | Yes | | The sample included all participants who took part in the workshop |
| 2 | | | Yes | | The workshop participants |
| 3 | | | Yes | | See Tables 3 and 4 |
| 4 | | | Yes | | There is complete data from the cases |
| 5 | | | Yes | | The data were analyzed using qualitative and quantitative methods. Participant  perceptions, experiences, areas needing strengthening and obstacles to ODS were  analyzed using manual content analysis (Berelson, 1971). Descriptive categories  were formed from the content of focus group discussions after they were transcribed and significant ideas extracted. The relevant categories were linked and themes identified. Quantitative data were analyzed using numbers, percentages, means and standard deviations. The analysis was conducted using the SPSS statistical package. |
| **24** | | | **Study ID: 2509**  **Author and Year: Kamiru …. (2009)**  **Study Design: Mixed Methods**  **Overall Score: 5** | | |
| S1 | | | Yes | | To evaluate Baylor International Pediatric AIDS Initiative’s (BIPAI) HCP training program in Swaziland. |
| S2 | | | Yes | | A multimethod design with two types of data collection and analysis: (1) one-group pretest-posttest survey with 101 HCPs; and (2) semi-structured in-depth interviews with seven trainers from Baylor College of Medicine and 16 local HCPs in Swaziland. |
| 1 | | | Yes | | This paper triangulates information from two sources to describe the effectiveness of a training program to enable HCPs in Swaziland to implement ART to pediatric patients. First, we analyzed data from a pre and post training questionnaire administered, before and after the training. After the training, semistructured in-depth interviews were conducted with program trainers and trainees to assess program coverage and delivery. |
| 2 | | | Yes | | Analyzed data from a pre and post training questionnaire administered before and after the training and qualitative to assess program coverage and delivery. Therefore it was coverage, delivery and impact. These themese were reported sequentially and integrated in the discussion. |
| 3 | | | Yes | | The themes were interpreted and integrated in the findings and discussion sections |
| 4 | | | Yes | | No divergences reported. |
| 5 | | | Yes | | Full details are given in the methodology section and in the limitations section at the conclusion of the article. |
| **25** | | | **Study ID: 2506**  **Author and Year: Kim et al 2009**  **Study Design: Quantitative Non-Randomised Study**  **Overall Score: 3** | | |
| S1 | | | Yes | | To evaluate the effectiveness of ‘village health worker training program‘ which aimed to build community participatory health promotion capacity of community leaders in villages of LMICs and to develop methods for further development of the program. |
| S2 | | | Yes | | Pre-post assessment |
| 1 | | | Yes | | 25 villages with populations more than 300 and without medical facilities and workers were selected. Among the population who can read and write in Tagalog at the ages between 15 and 55, 5 to 7 subjects were selected per Barangay as recommended by the PBCC and a total of 150 subjects were prepared. |
| 2 | | | Yes | | All listed in Table 2 |
| 3 | | | CT | | Cannot tell |
| 4 | | | CT | | Cannot tell |
| 5 | | | Yes | | There is no detail to say otherwise |
| **26** | | **Study ID: 3480**  **Author and Year: Kohrt et al (2020)**  **Study Design: Mixed Methods**  **Overall Score: 5** | | | |
| S1 | | Yes | | To evaluate a co-facilitation the RESHAPE (REducing Stigma among HealthcAre ProvidErs) intervention embedded within mhGAP training in Nepal. | |
| S2 | | Yes | | Data from observation, Likert Scales and MCQs. | |
| 1 | | Yes | | The study and it’s components are set up in great detail and well-intergrated. | |
| 2 | | Yes | | There is strong integration. | |
| 3 | | Yes | | The interpretation is adequate | |
| 4 | | Yes | | There is a full account of how the findings relate | |
| 5 | | Yes | | There is sufficient detail on the underlying procedures for each component of the study. | |
| **27** | | | **Study ID: 2489**  **Author and Year: Mazia et al (2009)**  **Study Design: Quantitative Non-Randomised**  **5Overall Score: 5** | | |
| S1 | | | Yes | | To evaluate the effectiveness of the new PNC services. |
| S2 | | | Yes | | A quasi-experimental prepost-test design |
| 1 | | | Yes | | The sample size was determined using monitoring data |
| 2 | | | Yes | | Measures are commonly used indicators of ANC service use and included quantitative and qualitative (observations) |
| 3 | | | Yes | | Data is presented for the full sample |
| 4 | | | Yes | | Necessary steps were taken to reduce bias. |
| 5 | | | Yes | | The intervention was administered as intended. |
| **28** | | | **Study ID: 210**  **Author and Year: McConnell … (2017)**  **Study Design: Quantitative Descriptive Study**  **Overall Score: 5** | | |
| S1 | | | Yes | | To strengthen a long-term health professional capacity building partnership between Guatemalan and US-based partners. |
| S2 | | | Yes | | Evaluation of the program consisted of pre- and post-knowledge assessments by the learners as well as quality and satisfaction evaluations by both learners and instructors |
| 1 | | | Yes | | Workshop Participants |
| 2 | | | Yes | | Workshop Participants |
| 3 | | | Yes | | Pre and post knowledge assessments, scores provided in Figure Two |
| 4 | | | Yes | | All participants completed the assessments |
| 5 | | | Yes | | Percentage increase |
| **29** | | **Study ID: 4466**  **Author and Year: Mehrotra et al (2018)**  **Study Design: Mixed Methods**  **Overall Score: 4** | | | |
| S1 | | Yes | | To ascertain the effectiveness of Project ECHO, a Hub and Spokes tele-mentoring model to bridge the urban-rural divide in mental health and addiction care. | |
| S2 | | Yes | | Data collected through a needs assessment, surveys and learning assignments | |
| 1 | | Yes | | There are justifications for each approach used and their integration | |
| 2 | | Yes | | The components are presented sequentially within the findings and integrated in the discussion | |
| 3 | | Yes | | Strong interpretation is provided in the analysis | |
| 4 | | Yes | | There is a full account of how the findings relate | |
| 5 | | No | | Some details are absent | |
| **30** | | | **Study ID: 1049**  **Author and Year: Merchant …. (2015)**  **Study Design: Quantitative Descriptive Study**  **Overall Score: 5** | | |
| S1 | | | Yes | | Training Laypersons and Hospital Personnel in Basic Resuscitation Techniques: |
| S2 | | | Yes | | A pre-test intervention, and post-test to evaluate and demonstrate first response skills. |
| 1 | | | Yes | | Training participants |
| 2 | | | Yes | | Eighty-eight people of 100 completed both the pre- and post-tests. |
| 3 | | | Yes | | Test scores |
| 4 | | | Yes | | Training participants |
| 5 | | | Yes | | A t test for equal variances demonstrated significant difference between the post-intervention scores for the two groups (p = 0.01). All 100 participants were able to open an airway, externally control haemorrhage, and transport a patient with appropriate precautions. |
| **31** | | | **Study ID: 2084**  **Author and Year: Minh et al (2013)**  **Study Design: Quantitative non-randomized study**  **Overall Score: 4** | | |
| S1 | | | Yes | | To assess the effectiveness of a training and supportive supervision intervention in strengthening the capacity of pharmacy staff in Vietnam |
| S2 | | | Yes | | Pre- and post-intervention study using a cross-sectional design. |
| 1 | | | Yes | | A simple random sampling technique was applied to identify subjects for pharmacy staff surveys, in which the sampling unit was an individual staff member. The minimum sample size was calculated using WHO’s recommended formula (Lwanga & Lemeshow 1991) to detect the difference in staff knowledge before and after the  interventions at a level of significance (a) of 5% and power of test (1- b) of 80%. The calculated sample size was 270; a 4% non-response rate for the baseline survey  and 2% for the post-intervention survey were taken into account, giving a final sample size of 281 and 275 for the two surveys, respectively. |
| 2 | | | Yes | | A range of measures on knowledge, attitudes and practice |
| 3 | | | Yes | | Pre-intervention, n = 281. Post-intervention, n = 275 |
| 4 | | | No | | No details given |
| 5 | | | Yes | | There is no account of any deviation or intrusion. |
| **32** | | | **Study ID: 2482**  **Author and Year: Murugesan et al (2009)**  **Study Design: Quantitative Descriptive**  **Overall Score: 4** | | |
| S1 | | | Yes | | To evaluate the impact of a diabetes training programme for PCPs |
| S2 | | | Yes | | Impact of training was assessed by pre- and post-training knowledge scores, feedback on usefulness of training modules, prioritizing activities to be introduced  in their practice and methods to be used for raising public awareness on diabetes. |
| 1 | | | Yes | | Training participants |
| 2 | | | Yes | | 2865 of 3023 participants responded to the evaluation questionnaire |
| 3 | | | CT | | Insufficient detail, cannot tell |
| 4 | | | Yes | | Responses collected |
| 5 | | | Yes | | z-test was used to compare the proportion of improvement in knowledge scores and for comparison of scores on various education modules. |
| **33** | | | **Study ID: 118**  **Author and Year: Mutale et al (2017)**  **Study Design: Mixed Methods (cross-sectional, mixed method study)**  **Overall Score: 5** | | |
| S1 | | | Yes | | An impact evaluation of the Zambian Management and Leadership Academy (ZMLA) programme conducted in 2014. |
| S2 | | | Yes | | A cross-sectional mixed method study. The study targeted health workers, stakeholders and course implementers. |
| 1 | | | Yes | | ZMLA trainees were targeted to gain perspectives on the extent to which the program affected levels of self-confidence resulting from knowledge gained. Perspectives were sought from both ZMLA and non-ZMLA trainees to measure changes in the work environment. Stakeholder perspectives were collected from trainers, and key informants involved in providing ZMLA training. |
| 2 | | | Yes | | The findings are discussed thematically, moving through the data sources and linking with coherence. |
| 3 | | | Yes | | Overall, the assumptions of the ZMLA theoretical framework were supported by the results. |
| 4 | | | Yes | | There are no details on inconsistencies |
| 5 | | | Yes | | A full account is given of the data management and analysis procedures |
| **34** | | | **Study ID: 1715**  **Author and Year: Namagembe et al (2012)**  **Study Design: Quantitative descriptive**  **Overall Score: 5** | | |
| S1 | | | Yes | | Evaluation of training to multidisciplinary teams of clinical, laboratory and health information assistants. |
| S2 | | | Yes | | Sample includes clinicians and laboratory professionals and health information assistants who were part of a training to improve management of febrile patients and encourage communication and trust. |
| 1 | | | Yes | | They sampled all from the pool of trained professionals. |
| 2 | | | Yes | | All sampled were those trained |
| 3 | | | Yes | | The measures to understand clinical and laboratory skill were matched to the profession and training |
| 4 | | | Yes | | While not stated explicitly, from table 1 can be seen that of 118 trained, 101 were observed. |
| 5 | | | Yes | | This is described and documented, and relevant to the research question |
| **35** | | | **Study ID: 1025**  **Author and Year: Namazzi et al… (2015)**  **Study Design: Mixed Methods**  **Overall Score: 4** | | |
| S1 | | | Yes | | Aimed to increase frontline health worker capacity through district-led training, support supervision, and mentoring at one district hospital and 19 lower-level facilities. |
| S2 | | | Yes | | Quantitative data collection was done within routine process monitoring and qualitative data were collected during support supervision visits |
| 1 | | | Yes | | To document implementation experiences and contextual factors and events (e.g. new facility management, major donor input to a facility or community) that might influence study outcomes. |
| 2 | | | Yes | | All analysis is integrated under the WHO HS Building Blocks framework |
| 3 | | | Yes | | They are interpreted adequately in the findings and discussion. |
| 4 | | | Yes | | None are disclosed |
| 5 | | | No | | Full and sufficient detail is not provided |
| **36** | | | **Study ID: 1005**  **Author and Year: Okereke et al (2015)**  **Study Design: Quantitative Descriptive**  **Overall Score: 4** | | |
| S1 | | | Yes | | This study determined if there were benefits and increases in knowledge levels for 33 selected health workers across 5 health facilities in Jigawa State following the introduction of clinical mentoring. |
| S2 | | | Yes | | Questionnaires were used to determine biodata and knowledge scores of mentored health workers and also key departmental activities before and after a 6 months period of introduction of clinical mentoring. |
| 1 | | | Yes | | Purposive sampling technique was used to recruit clinical mentors through the professional network of PATHS2's consultant on clinical mentoring from among  consultant obstetricians and consultant pediatricians working in Kano and Bauchi States (three consultant obstetricians and three consultant paediatricians).  33 mentees were randomly selected medical officers, nurses/midwives and community health extension workers (CHEWS) working in five selected health  facilities in Jigawa State |
| 2 | | | CT | | Nothing is said about this |
| 3 | | | Yes | | Knowledge scores on maternal and child health: areas of maternal, newborn and child health such as the use of magnesium sulphate for eclampsia management, causes of anemia in pregnancy, management of a patient with postpartum hemorrhage, management of normal labour as well as questions around obstetric emergencies, newborn resuscitation and infection prevention |
| 4 | | | Yes | | 33 trainees responded |
| 5 | | | Yes | | T-test statistical analysis of the pre- & post knowledge test scores of the 33 mentored health workers across the five clinical mentoring health facilities. |
| **37** | | **Study ID: 3418**  **Author and Year: Oladele et al (2020)**  **Study Design: Mixed Methods**  **Overall Score: 4** | | | |
| S1 | | Yes | | To improve health-care provider (HCP) awareness and clinical skills in the management and prevention of cryptococcal meningitis (CM). | |
| S2 | | Yes | | A 14-point pre and post-test assessment instrument was designed to capture the impact of the training and focus group discussions (FGDs) were conducted. | |
| 1 | | No | | No rationale is provided | |
| 2 | | Yes | | The different components are analysed and discussed in an integrated manner. | |
| 3 | | Yes | | Strong interpretation is provided in the analysis | |
| 4 | | Yes | | There is a full account of how the findings relate. | |
| 5 | | Yes | | A full account of procedures is provided. | |
| **38** | | | **Study ID: 424**  **Author and Year: Oleribe et al (2018)**  **Study Design: Quantitative Descriptive**  **Overall Score: 0** | | |
| S1 | | | Yes | | Assess impact of training to develop sustainable capacity to tackle MDG 6 targets in West Africa through better postgraduate medical education. |
| S2 | | | Yes | | Pre- and post- test scores |
| 1 | | | CT | | There is no account of the sampling strategy |
| 2 | | | CT | | Workshop participants, but no account or representation |
| 3 | | | CT | | Knowledge scores on medical leadership, malaria, tuberculosis and HIV/AIDS but no specific details of measures |
| 4 | | | CT | | 703 doctors were trained but no detail on numbers who took test. |
| 5 | | | CT | | No significance level was given, or details of test used. |
| **39** | | | **Study ID: 624**  **Author and Year: Perrone et al (2016)**  **Study Design: Mixed Methods**  **Overall Score: 3** | | |
| S1 | | | Yes | | Evaluation of programme designed to teach leadership and management skills to laboratory supervisors, the programme enabled participants to improve laboratory testing quality and operations. |
| S2 | | | Yes | | Pre- post- scores and quizzes and assignments |
| 1 | | | Yes | | Surveys assessed learner satisfaction with content, and pre/post-course tests, and in-course quizzes and assignments measured participants’ comprehension; Capstone Project assignments demonstrated the application of course theory. |
| 2 | | | Yes | | Results are discussed together to offer an evaluation of the course |
| 3 | | | Yes | | Fully interpreted in the discussion |
| 4 | | | No | | No details of divergences are discussed. |
| 5 | | | No | | Insufficient details are provided |
| **40** | | | **Study ID: 948**  **Author and Year: Pringle et al (2015)**  **Study Design: Quantitative Descriptive**  **Overall Score: 4** | | |
| S1 | | | Yes | | A trauma curriculum tailored to low-resource settings was implemented in Managua, Nicaragua utilising traditional didactic methods and novel low-cost simulation methods. |
| S2 | | | Yes | | Knowledge gain in attending and senior residents was subsequently assessed by using pre- and postwritten tests, and by scoring pre- and post-simulation scenarios. |
| 1 | | | Yes | | The written exam questions and simulations were randomly assigned so that no questions or cases were repeated. |
| 2 | | | Yes | | A total of 33 participants attended the course, including 18 (55%) attending and 15 (45%) resident physicians, with a 97% completion rate. |
| 3 | | | CT | | Some details provided in Table 2 but nothing more specific |
| 4 | | | Yes | | All participants participated in the evaluation |
| 5 | | | Yes | | The Wilcoxon signed-rank test was used to compare pre- and post-training differences in the written exam, and the percentage of critical actions completed in simulations. Time to critical actions was also analyzed using descriptive statistics |
| **41** | | | **Study ID: 595**  **Author and Year: Scheel et al (2016)**  **Study Design: Quantitative Descriptive**  **Overall Score: 4** | | |
| S1 | | | Yes | | To determine whether a 2-day educational course using a condensed Breast Imaging Reporting and Data System (condensed BI-RADS) improved the accuracy of Ugandan healthcare workers interpreting breast ultrasound. |
| S2 | | | Yes | | A pretest knowledge assessment, a series of lectures on breast imaging interpretation and standardized reporting using a condensed BI-RADS, and a post-test knowledge assessment. |
| 1 | | | CT | | No details of sampling strategy given, but the target audience of this intervention was Ugandan healthcare workers involved in performing, interpreting, or acting on the results of breast ultrasound. Sixty-one Ugandan healthcare workers participated in this study, including 13 radiologists, 13 other physicians, 12 technologists, and 23 mid-level providers. |
| 2 | | | Yes | | Full course participants who were trained on the intervention were samples. |
| 3 | | | Yes | | A range of measures related to knowledge of BI-RADS are reported |
| 4 | | | Yes | | All course participants took part |
| 5 | | | Yes | | The average per cent correct for each occupation on the pre- and post-tests was compared. Significant changes in the average per cent correct were determined using a paired t test for finding type and McNemar test for (1) individual finding types; (2) descriptors for masses; (3) concordance between the BI-RADS assessment categories and the participants’ recommendations; and (4) biopsy recommendations for normal (BI-RADS 1), benign (BI-RADS 2), and probably benign (BI-RADS 3) findings. |
| **42** | | | **Study ID: 2833**  **Author and Year: Sennun et al (2006)**  **Study Design: Mixed Methods**  **Overall Score: 5** | | |
| S1 | | | Yes | | To compare and identify the strengths and challenges of two different supervision models, to determine the effects on enhancing the health promotion capacity of health officers in two PCUs in Chang Mai, northern Thailand. |
| S2 | | | Yes | | Questionnaires to assess job satisfaction, clients’ perceived service quality and care satisfaction; and semi-structured interview and qualitative observations to explore the involvement of health officers and the community, and to determine the strengths and challenges of each supervisory model. |
| 1 | | | Yes | | As above |
| 2 | | | Yes | | They are analysed sequentially and then integrated to provide a full analysis |
| 3 | | | Yes | | They are interpreted to answer the research question |
| 4 | | | Yes | | None are reported |
| 5 | | | Yes | | A full account of the quantitative and qualitative procedures are provided. |
| **43** | | **Study ID: 3576**  **Author and Year: Sharma et al (2019)**  **Study Design: Quantitative Descriptive**  **Overall Score: 0** | | | |
| S1 | | Yes | | To measure self-reported changes in knowledge and confidence of primary care physicians in management of hypertension and its complications | |
| S2 | | Yes | | Pre- post- tests collected as part of the course evaluation | |
| 1 | | CT | | After the completion of the course, a representative sample from the batch was selected and an evaluation study was conducted to gather participant feedback on the program, and the results of the evaluation have been encouraging. However, no details of the sampling strategy are provided. | |
| 2 | | CT | | It is stated that a representative sample was achieved but no details are given. | |
| 3 | | CT | | No details are given but the results given are that 92% of the participants perceived an improvement in their knowledge regarding hypertension and its complications  after the course. A total of 87% of participants reported an improvement in confidence in initiating pharmacotherapy in patients with hypertension and 61% stated that their confidence in evaluating patients for complications of hypertension had improved. | |
| 4 | | CT | | No details are provided | |
| 5 | | CT | | No details are provided | |
| **44** | | **Study ID: 3385**  **Author and Year: Sijbrandij et al (2020)**  **Study Design: Randomised Control Trial**  **Overall Score: 3** | | | |
| S1 | | Yes | | To evaluate the effectiveness of a one-day PFA training on the acquisition and retention of knowledge of appropriate responses and skills in the acute aftermath of adversity in Peripheral Health Units (PHUs) in post-Ebola Sierra Leone | |
| S2 | | Yes | | Data to answer the research question was collected via a range of measures. | |
| 1 | | CT | | Details on how randomisation was achieved are not provided. | |
| 2 | | Yes | | The baseline assessment took place in the period between March 6 to April 6 in 2017 | |
| 3 | | No | | 71 participants were not included in outcome data for the programme non-control group. | |
| 4 | | Yes | | Twelve trained assessors, who were blind to the group participants were assigned to, administered the instruments. | |
| 5 | | Yes | | Trainings showed acceptable-to-excellent fidelity with scores ranging from 72% to 95% adherence to key features of PFA training. | |
| **45** | | | **Study ID: 388**  **Author and Year: Soeters et al (2018)**  **Study Design: Quantitative Descriptive**  **Overall Score: 4** | | |
| S1 | | | Yes | | Assessment of IPC training provided to frontline healthcare workers (HCW) in healthcare facilities that were not Ebola treatment units, as well as to IPC trainers and IPC supervisors placed in healthcare facilities |
| S2 | | | Yes | | Trainings included both didactic and hands-on components, and were assessed using pre-tests, post-tests and practical evaluations. |
| 1 | | | No | | No sampling strategy is mentioned. 1625 people took part in trainings, in three prefectures in the forest region of Guinea. 49 and 55 supervisors and trainers also took part |
| 2 | | | Yes | | Infection prevention and control training scores |
| 3 | | | Yes | | The full response rates for all groups are noted in Table 2. |
| 4 | | | Yes | | Wilcoxon signed rank test with recorded p-values |
| 5 | | | Yes | | We calculated median percent increase in knowledge. |
| **46** | | **Study ID: 3376**  **Author and Year: Spagnolo et al (2019)**  **Study Design: Randomised Trial**  **Overall Score: 4** | | | |
| S1 | | Yes | | To evaluate the programme’s impact on PCPs’ mental health knowledge, attitudes, self-efficacy and self-reported practice, immediately following and 18 months after training. | |
| S2 | | Yes | | We conducted an exploratory trial with a combination of designs: a pretest–post-test control group design and a one-group pretest– posttest design were used to assess the training’s short-term impact; and a repeated measures design was used to assess the training’s long-term impact | |
| 1 | | Yes | | Sufficient detail is provided on processes of randomisation | |
| 2 | | Yes | | Full details on socio-demographic and practice characteristics are provided | |
| 3 | | Yes | | Outcome data is comprehensively presented in numerous tables, no reports of missing data. | |
| 4 | | Yes | | 112 PCPs were assigned specific ID numbers and randomized participants to either Group 1 or 2 using the Excel RAND function. Others involved in the trial were not informed of group allocation. | |
| 5 | | No | | Figure 4 presents recruitment and participation. Across Follow-ups and long term impact assessment, numerous participants were lost and/or declined to participate. However, researchers did assess potential bias due to drop-out, | |
| **47** | | | **Study ID: 91**  **Author and Year: Stephens et al (2017)**  **Study Design: Quantitative Descriptive**  **Overall Score: 2** | | |
| S1 | | | Yes | | To deliver and evaluate a short critical care nurse training course whilst simultaneously building local training capacity. |
| S2 | | | Yes | | Participant learning assessed through pre/post course Multi-Choice Questionnaires. |
| 1 | | | No | | No strategy but A total of 584 nurses and 29 faculty were trained. |
| 2 | | | No | | No, but it is reported to cover about 25% of the critical care nursing population |
| 3 | | | Yes | | MCQ Scores |
| 4 | | | No | | Table 2 shows the number of missing cases, which given the number could introduce nonresponse bias |
| 5 | | | Yes | | Univariate analysis was performed with standard descriptive statistics being used to describe the data. Wilcoxan signed-rank test (nonparametric) was used to compare pre and post MCQ results while independent sample t-test was used to compare parametric continuous variables. The level of significance set at p ≤ 0.05 |
| **48** | | **Study ID: 3979**  **Author and Year: Taieb et al (2018)**  **Study Design: Quantitative Descriptive**  **Overall Score: 2** | | | |
| S1 | | Yes | | To describe the level of knowledge and clinical practice regarding snakebite among health care staff. | |
| S2 | | Yes | | Yes, it quantified the attendees level of knowledge. | |
| 1 | | No | | There is no detail of a sampling strategy provided. | |
| 2 | | CT | | There are no details provided on whether the sample is representative of the wider population. Majority came from centre region, but unsure if this would mean not representative. | |
| 3 | | Yes | | 38 Multiple Choice Questions on the topic | |
| 4 | | No | | 63.3% of participants completed both pre- and post-test questionnaires. | |
| 5 | | Yes | | Characteristics of the attendees were compared between groups using the chi-2 or fisher test depending on the sample size for the categorical variables, and using the Wilcoxon nonparametric test for continuous variables. The score was compared between groups using the Wilcoxon non parametric test when two-groups were considered and the Kruskall-Wallis non parametric test otherwise. Pre- and post-training scores were compared using the Wilcoxon test for paired data. | |
| **49** | | **Study ID: 3543**  **Author and Year: Tarannum et al (2019)**  **Study Design: Quantitative Descriptive**  **Overall Score: 3** | | | |
| S1 | | Yes | | To scale up mental health services through capacity building of medical staff in refugee health facilities to enable them to identify and manage people with mental,  neurological and substance use conditions. | |
| S2 | | Yes | | Training evaluations and data from the refugee health information system and clinical supervision reports | |
| 1 | | Yes | | Purposive. Two to three primary health care workers from each UNHCR supported health facility were selected for mhGAP training: a total of 62 primary health care workers. The selection of trainees was done in close consultation with the health management of the facility, prioritising those staff who: (1) indicated they were interested in mental health and motivated use the skills in their daily work, (2) had work schedules that would allow participation in supervision sessions and (3) were likely to stay long term in their duty station. The majority (n=36) were doctors. Others were medical assistants (n=10), psychologists (n=6), health educators (n=7), medical coordinators (n=2) or nurses (n=1). | |
| 2 | | Yes | | All trainees were surveyed. | |
| 3 | | Yes | | Demonstration of skills and gain in knowledge | |
| 4 | | CT | | Response rates for presented data not always provided. | |
| 5 | | No | | No details of significance are provided | |
| **50** | | | **Study ID: 2392**  **Author and Year: Tharkar et al (2009)**  **Study Design: Mixed Methods**  **Overall Score: 3** | | |
| S1 | | | Yes | | To strengthen the capacity of primary care physicians in prevention and control of diabetes in Tamilnadu State in India. |
| S2 | | | Yes | | The impact of the training programme was evaluated in two parts – (i) knowledge assessment done by administering a tool to the doctors before the start and towards the end of the sessions and (ii) clinical practice assessment - interviewing the doctors to assess diabetes service delivery, after six months |
| 1 | | | No | | No explicit rationale is given |
| 2 | | | Yes | | A significant improvement in identification of risk factors and high risk groups, primary prevention methods, screening and diagnostic procedures and treatment of diabetes by the physicians were observed. After 6 months, considerable improvement in diabetic care delivery to patients at community level was observed and some physicians had started organizing screening and awareness campaigns in their communities. |
| 3 | | | Yes | | Knowledge assessment and clinical practice assessment results are clearly presented |
| 4 | | | Yes | | No divergences are reported |
| 5 | | | No | | A description of the evaluation is provided but no account of methodological principles are provided |
| **51** | | **Study ID: Manually retrieved through snowballing**  **Author and Year: Weaver, 2012**  **Study Design: Cluster Randomised Trial**  **Overall Score: 4** | | | |
| S1 | Yes | | | This study addresses the effect of a specific training on mid-level providers clinical competence for infectious disease management. | |
| S2 | Yes | | | It put forth two hypothesis that were then tested through a randomised cluster control trial, with methods specific to each. | |
| 1 | Yes | | | Randomisation was done at the level of health facility. Two arms, with 1:1 ratio. Figure 2 details process. Within these, participants were randomly assigned to three groups. | |
| 2 | Yes | | | No data provided to support this at results phase. However the detail provided on how randomization occurred indicates likelihood these groups are comparable. | |
| 3 | Yes | | | Data is complete and detailed. | |
| 4 | No | | | This study was not blinded. | |
| 5 | Yes | | | No large reports of participants deviating from intervention | |
| **52** | | **Study ID: 3956**  **Author and Year: Werdenberg et al (2018)**  **Study Design: Mixed Methods**  **Overall Score: 5** | | | |
| S1 | | Yes | | To describe ABC implementation outcomes, including development of a QI change package. | |
| S2 | | Yes | | A process evaluation of ABC implementation and its impact on healthcare worker (HCW) attitudes and QI practice was done using program documents, standardized surveys and focus groups with facility QI team members attending ABC Learning Sessions. | |
| 1 | | Yes | | The breadth of the study and it’s components is set up at the outset, with an implicit rationale. | |
| 2 | | Yes | | The different components are analysed and discussed in an integrated manner. | |
| 3 | | Yes | | Strong interpretation is provided in the analysis. | |
| 4 | | Yes | | The findings are multi-faceted. | |
| 5 | | Yes | | A full account of procedures is provided. | |
| **53** | | **Study ID: 1355**  **Author and Year: Williams et al (2014)**  **Study Design: Quantitative Descriptive**  **Overall Score: 5** | | | |
| S1 | | Yes | | To develop and sustain a national network of nurse-trainers who could provide ongoing HIV continuing education and training ex5periences to Vietnamese nurses. | |
| S2 | | Yes | | Data to assess the outcomes of the train-the-trainer workshops were collected using a confidential structured questionnaire. The questionnaire solicited professional demographic information, HIV knowledge, self-report of attitudes toward PLWH and injection drug users, self-confidence regarding ability to care for PLWH and injection drug users, and self-confidence regarding teaching skills. A quasi-experimental, one-group design was used to assess pre- and post-training changes in outcomes. Records regarding the number and types of programs conducted by the trainees between workshops were maintained by the trainees and reported quarterly to the program office. Finally, pre- and postworkshop changes on the part of participants in a subset of trainee-facilitated training programs were assessed with a structured questionnaire similar to the one used to assess outcomes of the train-the-trainer workshops. | |
| 1 | | Yes | | The initial cohort of trainees was selected in consultation with the Vietnam Nurses Association to fulfil the minimum criteria of including two nurses from each province, one with clinical responsibilities for the care of PLWH and the other with administrative and policy responsibilities at the provincial or city level. | |
| 2 | | Yes | | Since it is spread across provinces | |
| 3 | | Yes | | Knowledge scores reported in Table 2 | |
| 4 | | Yes | | Missing cases are recorded in Table 2 | |
| 5 | | Yes | | Scores were analysed using a quasi-experimental, one-group pre- and post-test design. Among the 1,052 participants completing the pre-workshop questionnaire for the basic HIV training courses, the mean knowledge score was 77 (range 5 15–100) of a possible perfect score of 100. Mean score for the post-workshop respondents (n 5 1,019) was 87 (range 5 15–100). The increase in scores was statistically significant (p 5 .001). | |
| **54** | | **Study ID: 3355**  **Author and Year: Wilson et al (2019)**  **Study Design: Mixed Methods**  **Overall Score: 3** | | | |
| S1 | | Yes | | To evaluate a CHW training programme in Malawi that integrated technology into rehabilitation care delivery. | |
| S2 | | Yes | | The data collected included the following: a written pre- and post-knowledge test, skills competency checklist and a post-training programme survey (with narratives). | |
| 1 | | No | | No rationale was provided for using a mix of methods | |
| 2 | | Yes | | The results of both approaches are discussed sequentially | |
| 3 | | Yes | | The results are interpreted comprehensively | |
| 4 | | Yes | | The results are compared in their sequential presentation | |
| 5 | | CT | | Sufficient detail is not provided | |
| **55** | | **Study ID: 3905**  **Author and Year: Yu et al (2019)**  **Study Design: Mixed Methods**  **Overall Score: 3** | | | |
| S1 | | Yes | | To examine the effectiveness and cultural compatibility of a community health worker (CHW) training programme on water, sanitation and hygiene (WASH) in Haiti. | |
| S2 | | Yes | | Mixed-methods including pre- and post-course tests of WASH knowledge, focus group discussions and semi-structured observation. | |
| 1 | | No | | There is no rationale as to why mixed methods were selected. | |
| 2 | | Yes | | Data from questionnaires, interviews and observation were compiled to answer the research question. | |
| 3 | | Yes | | Data are fully interpreted. | |
| 4 | | CT | | There is no information about divergences or inconsistencies. | |
| 5 | | Yes | | There is sufficient detail on the quality criteria of each component method. | |
