# Supplementary material for: What do we mean by individual capacity strengthening for primary health care in low- and middle-income countries? A systematic scoping review to improve conceptual clarity
Source: Hum Resour Health. 2021 Jan 6;19:5. doi: 10.1186/s12960-020-00547-y (PMC7789571; doi:10.1186/s12960-020-00547-y)
Supplement: Supplementary file 4 — Additional file 4: Pre-existing validated tools. [file 12960_2020_547_MOESM4_ESM.docx]

**Additional file 4: Validated Tools Included in the Review**

| **Title** | **Domain** | **Source** |
| --- | --- | --- |
| Risk Tool to calculate CVD score | Knowledge and Skills | Abrahams-Gessel, 2015 |
| Namibia Project ECHO consortium developed the 25-question clinical knowledge assessment using the MoHSS core competencies for clinical providers (physicians and nurses), the fifth edition of the Namibian National HIV Treatment Guidelines | Knowledge | Bikinesi et al, 2020 |
| The protocol for this trial and supporting CONSORT checklist are available as supporting information in the Weaver et al, 2012 publication; see Protocol S1 and Checklist S1 above under Weaver et al, 2012. | Practice | Burnett et al, 2018 |
| Maternal and Child Health Initiative | Knowledge and Skills | Dawson et al, 2016 |
| Validated instruments to measure clinical practices, quality assurance initiatives and workplace policies.  Two dimensions of HIV stigma and discrimination were assessed [84]: stigmatising by nurses against people with HIV (10 items) and stigmatising by co-workers and the community against nurses who provide care to people with HIV (9 items).  62: Davies B, Edwards N, Ploeg J, Virani T. Insights about the process and impact of implementing nursing guidelines on delivery of care in hospitals and community settings. BMC Health Serv Res. 2008; 8: 29.  72: Edwards N, Davies B, Ploeg J, Virani T, Skelly J. Implementing nursing best practice guidelines: impact on patient referrals. BMC Nurs. 2007;6:4.  83: Edwards N, Davies B, Danseco E, Brosseau L, Pharand D, Ploeg J, et al. Evaluation of nursing best practice guidelines: clinical management, quality assurance and referrals. Ottawa: University of Ottawa School of Nursing; 2005. Oct. Report No.: CHRU & NBPRU Publication No. M05-1.  In relation to capacity specifically, participants self-assessed on 7 items at the conclusion of the programme. These were: appraising existing evidence and identifying gaps; initiating and undertaking an evaluation project; ability to disseminate findings; valuing policy relevance and access; confidence to communicate to decision-makers; valuing contributions from people in different roles and levels; leadership and team skills to improve the health system. | Knowledge, Skills, Ability, Confidence | Edwards et al, 2016 |
| A MHL survey previously used in rural India (Kermode, Bowen, Arole, Joag, et al., 2009; Kermode, Bowen, Arole, Joag, & Jorm, 2010; Kermode, Longleng, Singh, Bowen, & Rintoul, 2009) and in Australia (Jorm et al., 1997; Kitchener & Jorm, 2002), including with migrant communities (Lam, Jorm, & Wong, 2010; Minas, Colucci, & Jorm, 2009).  Stigmatizing attitudes were assessed using the Stigma Questionnaire (SQ) (Jadhav et al., 2007). | Knowledge, Attitudes and Confidence | Hofmann-Broussard et al, 2017 |
| The full protocol for the Trial Design is available as a supplementary file for Mbonye et al. and Weaver et al. The CONSORT Checklist for the trial is in Additional file 1 of this publication.  A pretested standardized assessment tool was used to record trainees’ clinical practice and anonymous patient information on these six sets of tasks. The tool was based on previous IMCI and JUMP evaluation tools with two important innovations from the work of Brentlinger et al. | Knowledge and Practice | Imani et al, 2015 |
| Service Provider Survey Questionnaire | Knowledge | Kabir and Hossain, 2019 |
| The self-efficacy scale used in this study has been used in the Nigerian intervention trial to increase HCPs’ comfort in providing care to HIV patients (Ezedinachi et al., 2002).  Attitudes toward AIDS were assessed using a 12-item scale (Trochim, 2004). | Knowledge, Attitude and Self-Efficacy | Kamiru et al, 2009 |
| Mental Health Global Action Programme Knowledge Assessment  Mental Health Global Action Programme Attitude Scale  Social Distance Scale (select components of Stigma in Global Context – Mental Health Study  ENACT Enhancing Assessment of Common Therapeutic Factors | Knowledge, Attitude, Competence | Kohrt et al, 2020 |
| ‘Health Promotion Capacity Checklist’ by Prairie Region Health Promotion Research Center. | Knowledge, Skills, Commitment, Resources | Kim et al, 2009. |
| USAID/BASICS, was an ongoing monitoring of quality of care. | Knowledge, Skills and Competencies | Mazia et al, 2009 |
| The questionnaire and scenario for ECP were adapted from another PATH project (PATH 2006) that had been used in Vietnam and several other countries (Cambodia, Nicaragua and Kenya). | Knowledge, Attitude and Practice | Minh et al, 2013 |
| Adapted from the Clinical Leadership Competency Framework (CLCF) and Medical Leadership Competency Framework (MLCF) of the United Kingdom. (www. leadershipacademy.nhs.uk). All self-rated. | Knowledge, Self-Efficacy and Motivation | Mutale et al, 2017 |
| Professional Quality of Life Scale (ProQOL-5) | Professional Quality of Life | Sijbrandj et al, 2020 |
| A 30-question, multiple-choice test regarding Ebola and IPC practices (S1 Appendix) was developed by staff from the Guinea Ministry of Health and Public Hygiene, WHO, INSP, and CDC | Knowledge | Soeters et al, 2018 |
| Mental Health Global Action Programme Knowledge Assessment  The Mental Illness: Clinicians’ Attitudes (MICA) Scale (version  4.0) | Knowledge, Attitude | Spagnolo et al, 2020 |
| Mental Health Global Action Programme Humanitarian Intervention Guide (mhGAP – HIG) | Skills | Tarranum et al, 2019 |
| The protocol for this trial and supporting CONSORT checklist are available as supporting information; see Protocol S1 and Checklist S1 within the publication | Knowledge and Skills | Weaver et al, 2012 |
